# Progress and Challenges of Messenger RNA Vaccines in the Therapeutics of NSCLC

**DOI:** 10.3390/cancers15235589

**Published:** 2023-11-26

**Authors:** Eftychia Kiousi, Vasiliki Lyraraki, Georgia Lamprini Mardiki, Nikolina Stachika, Aikaterini Konstantina Damianou, Christina Panagiotis Malainou, Nikolaos Syrigos, Georgia Gomatou, Elias Kotteas

**Affiliations:** Oncology Unit, Third Department of Medicine, Sotiria General Hospital, National and Kapodistrian University of Athens, 11527 Athens, Greecenksyrigos@med.uoa.gr (N.S.); georgiagom@med.uoa.gr (G.G.)

**Keywords:** messenger RNA, mRNA vaccines, anticancer vaccines, NSCLC, lung cancer

## Abstract

**Simple Summary:**

Currently, cancer immunotherapy is rapidly developing, including the use of monoclonal antibodies, adoptive T-cells, and mRNA vaccines. Due to their extensive use during the COVID-19 pandemic, mRNA vaccines are proving to be a promising therapeutic strategy for various medical fields, such as oncology. A number of clinical trials aim to evaluate the safety and efficacy of these vaccines in the treatment of solid tumors. Several studies are in the process of investigating mRNA vaccines when combined with other immunotherapeutic drugs and methods. Research has yet to provide practice-changing results considering NSCLC. However, based on the existing outcomes of the ongoing studies, an increase in clinician awareness of this therapeutic approach is significantly important. Therefore, the goal of this review is to highlight the progress and obstacles concerning the development of mRNA vaccines for the treatment of NSCLC and present ways in which they could be used in clinical practice.

**Abstract:**

The introduction of immune checkpoint inhibitors in the therapeutics of non-small cell lung cancer (NSCLC) has been a game-changer in the management of patients with lung cancer; however, challenges do exist since a non-negligible subset of patients does not respond to therapy. Various immunotherapeutic anticancer strategies have been increasingly developed in recent years, including monoclonal antibodies, adoptive T-cell therapy, and vaccines. Fueled by their rapid drug development and successful implementation during the COVID-19 pandemic, messenger RNA (mRNA) vaccines represent an emerging therapeutic approach in other fields of medicine, including oncology. Several clinical trials are currently being conducted to assess the safety and efficacy of mRNA vaccines regarding a variety of solid tumors. Combining mRNA vaccines with other immunotherapeutic approaches has also been suggested and is currently under investigation. Although, in the case of NSCLC, the investigation is still in its early stages, the initial results raise the need for clinician awareness of these promising therapies. To this end, in the present review, we aim to summarize current advances in the development of mRNA vaccines in NSCLC therapeutics and discuss pragmatic challenges regarding their drug development and the different opportunities for implementation.

## 1. Introduction

The introduction of immune checkpoint inhibitors (ICIs) in the therapeutics of non-small cell lung cancer (NSCLC) during the last ten years has been a game-changer in the management of patients with lung cancer, leading to durable responses and significant survival benefits [1,2,3,4]. Favored by its immunogenic nature, NSCLC has been a successful paradigm of the incorporation of ICIs in the therapeutic armamentarium of the disease, starting from the advanced stages, and was recently launched in the management of early NSCLC [5,6]. The standard of care for early-stage non-oncogenic-driven NSCLC is surgical resection with (neo)adjuvant chemotherapy and/or immunotherapy in high-risk patients. Locally advanced disease is treated with chemo-radiotherapy and maintenance immunotherapy, while the standard of care for metastatic disease includes combined chemo-immunotherapy or ICI monotherapy in selected cases [1,2,3,4,5,6]. Nevertheless, challenges associated with ICIs do exist, with a subset of patients suffering from toxicities, termed immune-related adverse events (irAEs) [7,8]. The manifestation of irAEs is unpredictable and may involve any system of the human organism [8]. The majority is manageable via withholding immunotherapy and using immunosuppressive agents. Life-threatening irAEs may rarely present, such as pneumonitis and neurological irAEs [7,8]. In addition, another major challenge is that a non-negligible proportion of patients exhibit resistance to ICIs that is either primary or acquired [9,10]. 

It should be noted that apart from ICIs, other modalities of immunotherapy have been developed in anticancer research with the aim of activating the host’s antitumor immunity, modifying the suppressive tumor microenvironment (TME), and ultimately improving the anticancer response, including T-cell therapy and vaccines [11,12,13]. In particular, immunotherapeutic strategies that use vaccines as vectors of cells, peptides, viruses, and nucleic acids have been proposed as an attractive option with both prophylactic and therapeutic potential [12,14]. 

Anticancer vaccines with prophylactic intent have mainly been developed in the case of a known causative agent, the highlight being the human papillomavirus (HPV) vaccines that provide high levels of protection against cervical cancer and other HPV-related neoplasms [15]. Such vaccines prevent infection by an oncogenic virus; however, they have limited effectiveness against already-developed tumors. On the other hand, therapeutic anticancer vaccines have been under investigation for many years but are without clinical success due to a plethora of challenges, primarily of a pharmaceutical technology nature [16]. Such challenges mainly involve an optimal vaccine formulation in order to be safe and concomitantly effective [16]. Notably, renewed interest has been raised in vaccines, fueled by the success of messenger RNA (mRNA) vaccines against SARS-CoV-2 [17]. The rationale of mRNA technology—which was already being investigated in anticancer research before the outbreak of the pandemic—is to use synthetic mRNA strands (either naked or connected with other molecules) encoding cancer cell proteins, the expression of which elicits immune responses against tumor antigens, including antibodies and cytotoxic T-cells [18]. Unlike other vaccines, the production of mRNA-based vaccines is relatively simple [12]. However, the efficient delivery of mRNA in vivo was considered a major challenge, limiting their clinical application for many years [12]. In recent years, significant advances in their delivery systems have been made, enabling their broader investigation. 

The emerging and widespread use of mRNA technology vaccines has boosted many clinical trials regarding their use in cancer immunotherapy [16,19]. Although most of these studies are in the early clinical phases, their initial results are encouraging and raise the need for clinician awareness of these promising therapies. Very recently, a phase III clinical trial was launched using an mRNA vaccine in combination with pembrolizumab in patients with resected melanoma, following a positive phase 2b trial [20]. With regard to NSCLC, the development of mRNA vaccines is in earlier steps. The present review aims to summarize the available evidence from clinical trials and recent advances in preclinical research to demonstrate the role of mRNA vaccines in NSCLC therapeutics and inform clinicians of the upcoming challenges.

## 2. Materials and Methods

Firstly, a comprehensive search in the clinicaltrials.gov database was performed (accessed on 25 August 2023). The clinical trials were retrieved from the clinicaltrials.gov database using the following query: “lung cancer AND mRNA vaccines”. All clinical trials, either completed or ongoing, related to the therapeutic treatment of NSCLC based on mRNA vaccines were included. Additionally, a literature review was performed in the PubMed database with combinations of the following keywords “cancer immunotherapy”, “mRNA vaccine”, “lung cancer”, and “NSCLC”. Non-English literature was excluded. Among the retrieved abstracts, we included recent (last three years) preclinical studies regarding the investigation of mRNA vaccines in the context of NSCLC. This search was followed by a manual search of the reference list of the included articles to identify additional studies. Finally, we included representative comprehensive reviews on the topic in order to highlight the background research in this field.

## 3. Pharmaceutical Development of mRNA Vaccines

The main types of anticancer vaccines are cell-based, protein/peptide-based, viral vectors, and gene-based vaccines (either DNA or RNA). Using mRNA in vaccines, despite its theoretical advantages, such as easy and cost-effective laboratory production and direct translation to protein in the cytoplasm without entering the nucleus, was long considered a challenging approach due to the molecule’s instability [21,22]. Improvements in the stability, translation efficiency, and innate immunogenicity of mRNA were expedited by applying optimal RNA nucleoside modifications affecting the coding sequence, the 5′-untranslated region (UTR), and 3′-UTR [21,22]. Unmodified RNA chains are highly immunogenic and are prone to degradation before even being translated to the desired protein. The in vitro application of modifications before proceeding with the delivery, such as harboring pseudouridine and RNA methylation as well as adjustments in the 5′ cap structure and poly(A) tail, are associated with more effective and safe vaccination [21].

An additional issue of mRNA vaccine technology is that the elongated single-stranded chain of mRNA makes it difficult to achieve high cellular encapsulation efficiency and drug loading; thus, applicable formulation strategies are usually required for effective delivery [22]. In recent years, significant progress has been made, both in the delivery systems and in identifying the appropriate targeted tumor antigen(s) in order to optimize the immune response [16,23]. 

Regarding the delivery systems, the main platforms for mRNA vaccines in older studies were the dendritic cells (DCs) [24]. DCs are professional antigen-presenting cells (APCs) that are able to engulf cellular material from their surroundings and stimulate naïve T cells [23]. The ex vivo transfection of DCs with mRNA is preferred, compared to in vivo transfection [19]. However, ex vivo generation is arduous and time-consuming [23]. Although this platform is safe [23], efficacy is a major challenge since immune responses to these vaccines are not significantly induced. The mRNA transfection rate of DCs is unsatisfactory [24]. The co-transfer of DC vaccines with lysosomal compartments, interleukin 12 (IL-12), or cytokines could increase their immunogenicity [23]. Lipid-based formulations are an alternative delivery system that circumvents the necessity of DC isolation, ex vivo cultivation, and re-infusion. Lipid nanoparticles (LNPs) consist of an ionizable amino-lipid-like molecule, lipid-anchored polyethylene glycol (PEG), a helper phospholipid, and cholesterol [12,19]. At physiological pH, ionizable lipids improve stability and reduce cytotoxicity as they remain neutral. At low pH, ionizable lipids are positively charged and facilitate the encapsulation of the mRNA and its release from the endosome to the cytoplasm. They are characterized by high immunogenicity, rapid manufacture, and a high plasticity transfection rate [16]. The high inflammatory responses of LNPs explain their increased efficacy and, at the same time, the fact that they are relatively unstable [25]. In addition, protamines are cationic peptides that form complexes with mRNA through electrostatic interaction, protect mRNA from degradation by extracellular RNases, and can be combined with lipids. These complexes can also activate toll-like receptor 7/8 (TLR7/8) to elicit the T helper 1-type immune response [12]. Protamine is used as a stabilization agent for RNA delivery [26,27]. Protamine-based vaccines seem to be safe, feasible, well-tolerated, and cause mild adverse effects [23,27]. Another advantage of protamine-based platforms is their thermostability and their ability to be maintained without cold-chain storage. Nevertheless, they seem less effective compared to LNPs or other liposome-based platforms [27].

Furthermore, the primary goal of mRNA anticancer vaccines is to stimulate the immune response against cancer by harnessing one or multiple tumor antigens. Importantly, tumor antigens can be categorized into two main groups: tumor-associated antigens (TAAs), which are self-antigens that are abnormally expressed in cancer cells but also expressed in normal cells, and tumor neoantigens, which are the repertoire of peptides that are expressed in the surface of tumor cells but are not expressed in normal tissues and are recognized by antigen-specific T cell receptors (TCRs) through the cooperation of major histocompatibility complex (MHC) molecules [23]. Most developed mRNA vaccines encode either fixed tumor antigens or a personalized set of neoantigens [28]. Carcinoembryonic antigen (CEA), prostate-specific antigen (PSA), melanoma-associated antigen (MAGE) 1, survivin, tyrosinase, human telomerase reverse transcriptase (hTERT), and Wilms’ tumor 1 antigen (WT1) are among the most common specific encoded proteins used in mRNA vaccines [23]. However, this approach is often associated with insufficient immunogenicity and is hindered by the space–time heterogeneity of classic tumor antigens [28]. Personalized neoantigen-based therapies are increasingly employed, boosted by the application of bioinformatics technology, and are expected to overcome those obstacles. Theoretically, neoantigens are not subject to central immune tolerance and provide an opportunity for tailored immune activation [29]. Nevertheless, certain issues are yet to be investigated, such as the neoantigen prediction systems’ accuracy and the mRNA molecules’ encoding capacity for multiple antigens [30].

## 4. Clinical Trials of mRNA Vaccines Including Patients with NSCLC

The review of the trials revealed a number of studies with mRNA anticancer vaccines that were addressed either to patients with a variety of solid tumors, including NSCLC, or were addressed exclusively to patients with NSCLC. All the studies are phase I or II clinical trials. Details of the retrieved clinical trials are summarized in Table 1.

The trial known as NCT00004604 was conducted in the early 2000s and was designed to assess the safety and dose-limiting toxicity of a vaccine of DCs loaded with mRNA encoding for CEA. The study enrolled patients with CEA-expressing metastatic malignancies, including lung cancer [31]. More specifically, the phase I dose-escalation study included nine patients with CEA-expressing lung cancer. The results of phase I showed that no significant toxicities were observed, although all patients reported malaise and subcutaneous nodules at the site of injection. Therefore, it was demonstrated that the administration of CEA mRNA-transfected DCs was safe. The most feasible dose was 3 × 107 DC per injection. The responses were evaluated in 24 patients in phase I. There was 1 complete response (assessed by tumor marker), 2 minor responses, 3 with stable disease, and 18 with progressive disease [31]. A phase II trial followed this study, but it included only those patients with resected colorectal cancer [31].

Another phase I/II trial (NCT02688686) initiated in China was planned to assess the safety and efficacy of a DC vaccine combined with cytokine-induced killer (CIK) cells in patients with advanced NSCLC and bone metastases. The cells were transfected with adenovirus type 5 (Ad5) vectors containing mRNA molecules encoding for three fixed tumor antigens: suppressor of cytokine signaling (SOCS) 1, MUC1, and survivin. However, there are no published results from this trial. 

Another interesting concept involves trials investigating mRNA vaccines that target Kirsten rat sarcoma viral oncogene homolog (KRAS) mutations, which are frequent genetic alterations in several neoplasms. The study NCT03948763 is a phase I trial using an LNP-formulated mRNA vaccine (mRNA-5671/V941), either alone or in combination with pembrolizumab, in patients with KRAS-mutant NSCLC, colorectal cancer, or pancreatic adenocarcinoma. The vaccine is tetravalent and includes mRNA molecules corresponding to G12D, G12V, G13D, or G12C driver mutations in the KRAS gene, with the aim of enhancing antigen-specific T-cell responses following vaccination. The purpose of the study is to determine the recommended phase 2 dose. The study has been completed with an enrollment of 70 patients, but the results are not published yet. Similarly, the NCT05202561 trial is currently recruiting patients with advanced malignancies and KRAS mutations (G12C, G12D, or G12V), including lung cancer. This is a phase I single-arm, open-label study to evaluate the mRNA tumor vaccine’s safety, tolerability, antitumor activity, immunoreactivity, and pharmacokinetics. The vaccine’s safety is investigated either alone or in combination with a PD-1 inhibitor. 

Furthermore, CV9201 and CV9202 are two mRNA-based cancer vaccines developed for NSCLC, containing mRNAs encoding for a set of different tumor antigens to stimulate an adaptive cellular and humoral immune response. The NCT00923312 trial used CV9201, an mRNA-based vaccine with free and protamine-complexed full-length mRNAs that encodes for five NSCLC-related antigens: New York esophageal squamous cell carcinoma-1 (NY-ESO-1), melanoma antigen family C1 and C2, survivin, and trophoblast glycoprotein (5T4). A phase I/IIa dose-escalation trial was conducted, which enrolled 46 patients with locally advanced or metastatic NSCLC and who experienced at least stable disease after first-line treatment [32]. The patients received five intradermal CV9201 injections at different dosage levels. The assessment of safety was the primary objective, while secondary objectives included the estimation of T-cell responses against the five antigens and changes in immune cell populations. Regarding safety, the results demonstrated that all CV9201 dose levels were well-tolerated, and the recommended dose for phase IIa was 1600 µg. The reported AEs were mild injection-site reactions and flu-like symptoms. The immune response evaluation showed that CV9201 immunogenicity could be detected, but immune responses were relatively rare and were not persistent in nature, strongly indicating the need for improvement of the tested mRNA-immunotherapeutic [32].

In the trial NCT01915524, the researchers investigated whether a similar lung cancer vaccine (CV9202 or BI1361849) could be safely administered, in combination with local radiation therapy (RT), for the consolidation and maintenance of treatment of stage IV NSCLC after first-line chemotherapy or therapy with an EGFR tyrosine kinase inhibitor [33]. The vaccine comprised 6 mRNA molecules encoding for fixed tumor-associated antigens: Mucin1 (MUC1), survivin, NY-ESO-1, 5T4, MAGE-C2, and MAGE-C1, which are overexpressed in NSCLC compared to healthy tissue. Local RT is frequently used as a palliative treatment for metastatic lesions in the lung, bone, and soft tissue, and it was hypothesized that this could enhance the immunogenic effect of the vaccine. The vaccine was administered, along with maintenance chemotherapy when indicated. The regimen was well-tolerated according to the results; injection site reactions and flu-like symptoms were the most common AEs. Increased antigen-specific immune responses, including augmented antigen-specific antibody levels and functional T cells, were observed. Regarding tumor responses, 46.2% of the patients achieved stable disease (SD) as the best overall response, and one patient had a partial response (PR) in combination with pemetrexed maintenance [33].

The same vaccine (CV9202 or BI1361849) has been evaluated in an open-label, multicenter, two-armed study (NCT03164772) to assess the safety and preliminary efficacy of adding the vaccine to one or two ICIs for NSCLC, namely, the anti-PD-L1 antibody durvalumab and the anti-cytotoxic T-lymphocyte-associated protein 4 (CTLA-4) antibody tremelimumab [34]. The patients in arm A were treated with the mRNA vaccine plus durvalumab, while those in arm B received the vaccine plus durvalumab and tremelimumab. The evaluation of safety and tolerability was the primary endpoint, including dose-limiting toxicity during dose evaluation. Secondary endpoints were progression-free survival (PFS), the objective response rate (ORR), disease control rate (DCR), response duration, and overall survival (OS). Moreover, a control group treated with ICIs alone was added, in order to compare the immune responses [34]. This trial has been completed and 61 patients were enrolled in total, of which 24 were in arm A and 37 in arm B. Of these 61 subjects, 57 were treated with at least one dose of study treatment. Regarding toxicity, 56.5% in arm A and 55.9% in arm B presented treatment-related adverse events of any grade. In arm A, 26.3% presented PR as the best response, 36.8% SD, and 36.8% progressive disease (PD), while in arm B, the percentages were 11.1%, 29.6%, and 59.3%, respectively. It should be noted that the trial was not randomized.

Currently, the NCT03908671 trial is evaluating a personalized neoantigen mRNA vaccine. The vaccine is used as a monotherapy in patients with advanced esophageal cancer and NSCLC after the failure of standard treatment. The main objective of the trial is to evaluate the safety and tolerability of an mRNA personalized tumor vaccine and the secondary objective is the preliminary assessment of the efficacy of the vaccine in this patient population. The trial is currently enrolling patients. 

## 5. Recent Advances in Preclinical Research of mRNA Vaccines for NSCLC

One of the main challenges in cancer vaccines consists of identifying the relevant tumor antigens that will maximize the potential of the patient’s anticancer immune response and eliminate immune escape possibilities; therefore, several recent translational studies have focused on finding the optimal tumor antigens that could be used for mRNA vaccine development in NSCLC [35]. Those are primarily bioinformatics-based studies that used genomic data from publicly available datasets on genetic alterations for NSCLC [35]. Candidate targets are usually selected as those targets with a positive correlation with the filtration of immune cells and confirmed to induce APCs expression upon their appearance [35,36,37,38,39]. Researchers also seek to identify the expression patterns of NSCLC that might benefit from treatment with mRNA vaccination [35,36,37,38,39].

A set of lung adenocarcinoma-related genes, namely, GPRIN1, MYRF, PLXNB2, SLC9A4, TRIM29, UBA6, and XDH, were reported as candidate genes for mRNA vaccines in a recent study [35]. Xu et al. retrieved several candidate tumor antigens related to lung adenocarcinoma and with prognostic value and investigated their correlation with the expression of APCs [36]. The results revealed two genes, KLRG1 and CBFA2T3, as potential antigenic targets that could be used for mRNA vaccine development, specifically for lung adenocarcinoma [36]. Moreover, the data implied that early-stage tumors, with high immune cell infiltration and checkpoint expression and a low tumor mutation burden, might be suitable for mRNA vaccination [36]. In another study, five lung adenocarcinoma-related antigens (CCNB1, KIAA0101, PBK, OIP5, and PLEK2) were recognized as being significantly associated with immune infiltrating cells and were suggested as potential targets for mRNA vaccines. In the same study, the researchers suggested that immunologically “cold” clusters of lung adenocarcinoma, identified by their gene expression profiles, were the most appropriate phenotype for vaccination [37]. Another two genes with prognostic value, ZC3H12D and TXNDC5, were identified in a study by Zhao et al. as potential targets for lung adenocarcinoma vaccines, based on their correlation with APC infiltration and tumor purity [38]. Finally, in a study that focused on lung squamous carcinoma, it was demonstrated that the expression patterns of the genes bone morphogenetic protein 5 (BMP5) and claudin 5 (CLDN5) were positively correlated with antigen-presenting cell infiltration, indicating their potential for development as mRNA cancer vaccines [39].

Sun et al. selected a set of somatic genetic alterations after tissue-profiling a mouse lung cancer model and subsequently generated a neoantigen–RNA vaccine [40]. The vaccine was administered along with engineered T cells in the mouse model. The results demonstrated that the immune system can recognize lung cancer neoantigens and that the therapy was associated with a significant antitumor effect in mouse lung cancer [40]. Interestingly, an mRNA tumor vaccine was investigated in a recent preclinical study of a mouse model of lung cancer with bone metastasis [41]. More specifically, the vaccine included two molecules: an mRNA encoding for MAGE-A1, an immunogenic protein that is highly expressed in lung cancer and is involved in tumor proliferation, invasion, and metastasis [42], as well as monophosphoryl lipid A (mPLA), a toll-like receptor 4 (TLR4) agonist, which can be inserted into the hydrophobic layer of liposomes. The vaccine was delivered via nasal administration, due to the abundance of lymphoid tissue. It was observed that it stimulated the maturation of dendritic cells, polarized M2 macrophages into M1 macrophages, and cross-activated the innate and adaptive immune responses, as well as exerting antitumor activity by inhibiting the growth of metastatic tumors [41]. 

## 6. Discussion

The present literature review revealed that research on mRNA vaccines for NSCLC is in the early stages but has evolved in recent years. In the past decades, trials (NCT00004604, NCT02688686) mostly used mRNA vaccines encoding for a single, pre-fixed tumor antigen [31]. Gradually, mRNA vaccines encoding for multiple antigens [32,33] or with a personalized neoantigen approach (NCT03908671) have been introduced and explored. A notable trend in preclinical research can be observed with the aim of identifying suitable antigens specifically for the NSCLC subtypes of adenocarcinoma and squamous carcinoma [36,37,38]. In addition, it seems that the use of DCs for the delivery of mRNA is being abandoned, while nanoparticle lipid-based platforms are now being introduced (NCT03948763). The vaccines are investigated either as monotherapy or when combined with radiotherapy, chemotherapy, or ICIs, and also in different clinical settings, but these mostly involve metastatic NSCLC rather than its early stages.

All trials are in early phases (phase I and II), primarily evaluating the toxicity, establishing the recommended doses for further investigation, and assessing the immune responses of patients, as well as making a preliminary assessment of efficacy. Regarding toxicity, the adverse events associated with mRNA vaccines are mainly mild and include flu-like symptoms, fever, fatigue, diarrhea, and injection-site reactions such as erythema [31,33]. Immune responses have been detected, but comprehensive evaluations of the nature and duration of the responses are limited [32]. Antitumor activity in terms of responses and survival endpoints has been observed, but data are still too scarce to draw conclusions. Perhaps, novel clinical trial design and the implementation of predictive biomarkers will enhance the possibility of evaluating antitumor activity, even in the early stages of clinical investigation. Close collaboration with translational scientists is crucial. Overall, when reviewing twenty years of research in this field, the number of clinical trials seems limited. Also, research is not focused on a specific strategy; in contrast, several different strategies (regarding vaccine formulation or its use in combination with other agents) are being investigated. This might reflect the lack of benefit observed in the earlier trials, especially those using mRNA vaccines as monotherapy, which did not prompt the continuation of clinical development. Not all recent trials have been completed; therefore, their results are eagerly awaited.

The development of mRNA anticancer vaccines will likely expand because of their profound advantages [12]. It should be noted that the synthesis of mRNA in large quantities is more rapid and is simpler compared to peptide vaccines [22]. In addition, encoding the full length of a tumor antigen is possible with mRNA vaccines, unlike peptide vaccines, which may be shorter in length [43]. Thus, they allow the simultaneous presentation of multiple epitopes to APCs with both class I and II patient-specific human leukocyte antigen (HLA), offering less restriction by the human HLA types, which present heterogeneity across the human population [43]. The result is the stimulation of a broader T-cell response [44]. Compared to DNA, mRNA does not require insertion into the genome to be translated and is, therefore, considered safer and without oncogenic potential [22]. Challenges concerning stability and delivery have partially been overcome by applying RNA nucleoside modifications and enhancing the delivery systems [21].

With regard to NSCLC therapeutics in particular, a major issue is the identification of the most suitable phenotypes and the appropriate clinical scenario(s) so that mRNA vaccines might find their place in the armamentarium of the disease. Preclinical research has been focused on the prediction of the candidate targets of mRNA vaccines. These studies aim to guide the production of mRNAs that encode the corresponding proteins of the studied targets. Hence, mRNA molecules, coupled with efficient delivery platforms, could induce tailored T-cell responses. However, the candidate targets of mRNA vaccines are inconsistent among studies, indicating the need to validate the results of single studies [35,36,37,38]. In addition, preclinical research has focused on the recognition of distinct phenotypes, mostly based on immune-related expression profiles that could derive benefits from mRNA vaccines. Nevertheless, the results are ambiguous. Certain studies suggest that immunologically “hot” subtypes with high infiltration of T cells and the expression of immune checkpoints might benefit from mRNA vaccination [36,38]. In contrast, other studies propose that immunologically “cold” phenotypes might be more suitable for vaccination, given that vaccines provide an active immune response rather than releasing the brake, as is the case with ICIs [37]. In a recent preclinical study in small-cell lung cancer (SCLC), a cancer type that garners limited benefit from treatment with ICIs, it was also suggested that the immunologically “cold” subtype of SCLC, with an immunosuppressive tumor microenvironment and without immune-cell infiltration, might be the candidate phenotype for the application of mRNA vaccination [45].

**Figure 1:**
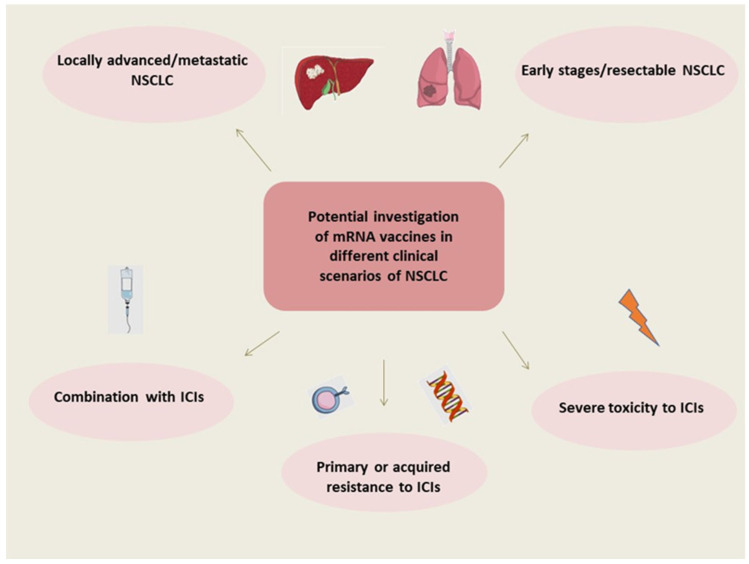
Potential investigation of mRNA vaccines in different clinical scenarios of NSCLC. Abbreviations: NSCLC: non-small cell lung cancer, mRNA: messenger RNA, ICIs: immune checkpoint inhibitors.

Finally, the optimal clinical scenario(s) to employ the mRNA vaccination of NSCLC has not yet been defined—and it might not be only one disease setting (as illustrated in Figure 1). Despite the great advances of recent years, the 5-year overall survival rate of advanced non-oncogenic-driven NSCLC is still dismal [3]. Also, approximately 40–50% of patients with early disease who are treated with surgery will relapse within 5 years [5]. Currently, mRNA vaccines are mostly investigated in the context of NSCLC, but the early disease setting should also be explored. It should be noted that the phase III mRNA vaccine clinical trial that was recently launched regarding melanoma involves patients with resected disease [20]. Investigation suggests that mRNA vaccines could be combined with other immunotherapeutic modalities. Furthermore, there are several scenarios of patients presenting resistance to ICIs who might require an alternative modality for stimulating the immune response or perhaps need re-sensitizing to treatment with ICIs [10,46], along with patients with comorbidities precluding treatment with ICIs [47,48,49,50], for whom a more specific and targeted immune activation could be investigated. Novel immune-related contributors, such as the gut and lung microbiome [51,52], could correlate with immune responses and might determine the suitable phenotypes to provide mRNA vaccination.

## 7. Conclusions

In conclusion, it seems that the development of mRNA vaccines for the therapeutic treatment of NSCLC is still in its infancy; however, clinical investigation and pre-clinical research have recently been enriched with new ideas on the topic. mRNA vaccination in NSCLC is being explored as a monotherapy or in combination with other therapeutic modalities. Establishing the optimal formulation of mRNA vaccines for NSCLC and solid tumors, in general, is a work in progress. Close collaborations between basic scientists and physicians, as well as between academia and industry, are warranted in order to maximize the potential new mRNA applications in thoracic oncology.

## Figures and Tables

**Table 1:** Completed or ongoing clinical trials of mRNA vaccines including patients with NSCLC.

NCΤ	Target Genes	mRNA	Delivery Platform	Trial Phase	Primary Endpoints	Secondary Endpoints	Status	N	Published Results [Ref]
NCT00004604	CEA	CEA RNA-pulsed DCs	DCs	I	Safety, Dose-limiting toxicity.	Immune response	Completed	24	Yes [31]
NCT02688686	SOCS 1, MUC1 and Survivin	Ad5vector-coding mRNAs	DCs, CIK	I/II	ORR	AEs	Unknown	30	No
NCT03948763	KRAS	mRNA-5671/V941	LNP	I	Dose-Limiting Toxicities, AEs	ORR, MutantKRAS Specific T cells	Completed	70	No
NCT05202561	KRAS	N/A	N/A	I	AEs	Antitumor activity immunoreactivity during treatment	Recruiting	10	No
NCT00923312	NY-ESO-1, MAGEC1/C2, survivin, and trophoblast glycoprotein	CV9201	Protamine	I/II	Recommended dose Safety and tolerability	Immune response Antitumor activity Correlation between TAA expression on tumor specimens and survival/progression/immunological response	Completed	46	Yes [32]
NCT01915524	MUC1, survivin, NY-ESO-1,5T4, MAGE-C2 and MAGE-C1	CV9202 or BI1361849	Protamine	I	≥grade 3 AEs	Immune response, overall tumor response, PFS, response to second-line cancer treatment, OS	Terminated	26	Yes [33]
NCT03164772	MUC1, survivin, NY-ESO-1,5T4, MAGE-C2 and MAGE-C1	CV9202 or BI1361849	N/A	I/II	AEs	ORR, PFS, duration of response, OS	Completed	61	Yes Available in clinicaltrials.gov
NCT03908671	N/A	Personalized neoantigen mRNA	N/A	N/A	AEs	DCR, PFS, TTP, OS	Recruiting	24	No

Abbreviations: DCs: dendritic cells; Ad5: Adenovirus 5; SOCS: suppressor of cytokine signaling; CIK: cytokine-induced killer; ORR: objective response rate; MUC1: mucin1; LNP: lipid nanoparticle; KRAS: Kirsten rat sarcoma viral oncogene homolog; NY-ESO-1: New York esophageal squamous cell carcinoma-1; MAGE: melanoma antigen family; TAA: tumor-associated antigen; TTP: time to tumor progression; DCR: disease control rate; PFS: progression-free survival; OS: overall survival; AEs: adverse events; NSCLC: non-small cell lung cancer; mRNA: messenger RNA.
